# Insulin-like growth factor 1 attenuates antiestrogen- and antiprogestin-induced apoptosis in ER^+ ^breast cancer cells by MEK1 regulation of the BH3-only pro-apoptotic protein Bim

**DOI:** 10.1186/bcr3153

**Published:** 2012-03-19

**Authors:** Sudharsan Periyasamy-Thandavan, Suchreet Takhar, Adam Singer, Michael Robert Dohn, William Hutch Jackson, April Eve Welborn, Derek LeRoith, Mario Marrero, Muthusamy Thangaraju, Shuang Huang, Patricia Veronica Schoenlein

**Affiliations:** 1 Department of Cellular Biology and Anatomy, Georgia Health Sciences University, 1459 Laney Walker Blvd., Augusta, GA 30912, USA; 2 Department of Cancer Biology, Vanderbilt University, 2220 Pierce Ave., Nashville, TN 37232, USA; 3 Division of Endocrinology, Diabetes and Bone Diseases, Mount Sinai School of Medicine, 1428 Madison Ave, New York, NY 10029, USA; 4 Institute of Vascular Biology, Georgia Health Sciences University, 1459 Laney Walker Blvd., Augusta, GA 30912, USA; 5 Department of Biochemistry, Georgia Health Sciences University, 1459 Laney Walker Blvd., Augusta, GA 30912, USA; 6 Medical College of Georgia Cancer Center, Georgia Health Sciences University, 1410 Laney Walker Blvd., Augusta, GA 30912, USA

## Abstract

**Introduction:**

In this pre-clinical *in vitro *study conducted in estrogen receptor positive (ER+) breast cancer cells, we have characterized the effects of insulin-like growth factor I (IGF-1) on the cytostatic and cytotoxic action of antiestrogen treatment when used as a single agent or in combination with the antiprogestin mifepristone (MIF). Our goal was to identify new molecular targets to improve the efficacy of hormonal therapy in breast cancer patients that have a poor response to hormonal therapy, in part, due to high circulating levels of unbound insulinIGF-1.

**Methods:**

IGF-1-mediated effects on cytostasis and apoptotic cell death were determined with cell counts conducted in the presence and absence of trypan blue; enzyme-linked immunosorbent assays to determine the intracellular levels of cleaved cytokeratin 18, a marker of epithelial cancer cell apoptosis; and immunoblot analysis to determine the levels of cleaved poly-ADP ribose polymerase (PARP) and lamin A that result from caspase-dependent apoptosis. Cytotoxicity was further characterized by determination of the levels of reactive oxygen species (ROS) and the percent of mitochondrial membrane depolarization in cell populations treated with the different hormones in the presence and absence of IGF-1. Small molecule inhibitors of the dual-specificity protein kinase MEK1, MEK1 siRNA, Bim siRNA, and vectors overexpressing MEK1 wild type and mutant, dominant negative cDNA were used to identify key IGF-1 downstream prosurvival effectors.

**Results:**

IGF-1, at physiologically relevant levels, blocked the cytotoxic action(s) of the antiestrogens 4-hydroxytamoxifen (4-OHT) and tamoxifen (TAM) when used as single agents or in combination with the antiprogestin MIF. The antiapoptotic action of IGF-1 was mediated primarily through the action of MEK1. MEK1 expression reduced the levels of ROS and mitochondrial membrane depolarization induced by the hormonal treatments via a mechanism that involved the phosphorylation and proteasomal turnover of the proapoptotic BH3-only Bcl-2 family member Bim. Importantly, small-molecule inhibitors of MEK1 circumvented the prosurvival action of IGF-1 by restoring Bim to levels that more effectively mediated apoptosis in ER^+ ^breast cancer cells.

**Conclusion:**

his study provides strong support for the use of MEK1 inhibitors in combination with hormonal therapy to effectively affect cytostasis and activate a Bim-dependent apoptotic pathway in ER^+ ^breast cancer cells. We discuss that MEK1 blockade may be a particularly effective treatment for women with high circulating levels of IGF-1, which have been correlated to a poor prognosis.

## Introduction

Breast cancer is a leading cause of cancer among women in the United States and approximately 60% to 70% of these breast cancers express estrogen receptor alpha (ERα) [1-3]. Estrogen binding to ERα induces both genomic and nongenomic actions of the ER, which ultimately lead to increased breast cancer cell growth. Over the past three decades, the selective estrogen-receptor modifier tamoxifen (TAM) has been used as an effective agent in adjuvant therapy and for the preoperative treatment for ER^+ ^breast cancer. TAM acts as a competitive inhibitor and prevents estrogen binding to the ER, blocking the proliferative and prosurvival effects of estrogen. However, only about two thirds of all ER^+ ^breast tumors are initially responsive to TAM therapy [4]. Moreover, the development of resistance to TAM and other antiestrogens occurs often in breast cancer patients and is a major clinical concern [3,5]. To understand the mechanisms of intrinsic and acquired resistance to antiestrogens, numerous *in vitr*o studies have been conducted, and the multiple mechanisms described by these studies have been reviewed [5,6]. However, it is still not clear which mechanisms commonly contribute to antiestrogen resistance in patients. Even with antihormonal therapies that severely deplete the estrogenic environment of the breast cancer cells, such as aromatase inhibitors, both inherent and acquired resistance occurs [7]. The fact that antiestrogen resistance is still a major obstacle to successful antiestrogen therapy underscores the importance of investigating new therapies or identifying effective combination therapies for the treatment of ER^+ ^breast cancer.

Because progesterone binding to the progesterone receptors (PRs), like estrogen binding to ERs, is growth stimulatory for breast cancer cells, using antagonists to both receptors to block tumor growth may be an attractive treatment option for ER^+ ^and PR^+ ^breast cancers. Such a combination therapy may be particularly applicable for breast cancer patients with PR A-rich tumors that typically show a poor disease-free survival rate [8]. MIF, also referred to as RU486, is the most commonly used antiprogestin. MIF effectively antagonizes the activities of the PR and has served as a prototype antiprogestin to block PR function in various breast cancer cell models used in preclinical *in vitro *studies [reviewed in [9]]. Recent studies using *in vivo *models have further established a growth-stimulatory role for progestins and an important antitumor role for MIF and other antiprogestins [10,11]. Further, a recent study on breast cell proliferation in premenopausal women provided evidence for a protective effect of MIF monotherapy on the breast epithelium through its ability to block breast epithelial cell proliferation [12].

*In vitro *studies conducted in ER^+ ^breast cancer cell models by our laboratory [11,13,14] and others [15,16] showed that the combination of an antiestrogen plus an antiprogestin induced significantly higher levels of cytostasis and cytotoxicity (cell death) than did treatment with the antiestrogen or antiprogestin used as a single agent. Our previous studies also showed superior efficacy of this combined treatment against antiestrogen-resistant, ER^+ ^PR^+ ^breast cancer cells in comparison to antiestrogen treatment [17]. Further, our studies provided strong evidence that the antiproliferative effects of MIF are mediated primarily via its binding to the PR and not via binding to the glucocorticoid and mineralocorticoid receptors [13].

In preclinical studies, MCF-7 cells, which express both ER and PR, have often served as the prototype ER^+ ^breast cancer model system. MCF-7 cells show E2-dependent growth and are growth stimulated by progestin binding to the PR [11,14,17]. In addition, IGF-1 stimulates the proliferation of MCF-7 cells, and cross-talk between ER and IGF-1R is required to stimulate maximal growth of MCF-7 cells [18]. IGF-1 binding to IGF-1R activates its tyrosine kinase activity and downstream signaling cascades [19], which include the phosphorylation and activation of MEK1/MAPK1/2 and PI3K/AKT signaling [20]. Activation of MAPK1/2, also referred to as extracellular signal-regulated-kinases ERK1 (p44) and ERK2 (p42), and AKT can ultimately increase breast cancer cell proliferation [21] and survival [22,23]. AKT-mediated signaling is viewed as a determinant in a breast cancer response to antiestrogen treatment [24]. A key role for AKT signaling in endocrine response is supported by the recent clinical study in which the targeting of mTOR, a downstream effector of AKT, sensitized ER^+ ^breast cancers to aromatase inhibitors [25]. MEK1/MAPK signaling also regulates cell growth and/or differentiation, but is not typically thought of as a key antiestrogen resistance mechanism or as a key effector of cell survival in breast cancer cells undergoing hormonal therapy [26]. However, MEK1 activation and subsequent phosphorylation of the MAPKs is associated with a poor response to antihormonal therapy and decreased patient survival in clinical breast cancer [27,28], and a recent study determined that blockade of MAPK affects co-repressor recruitment and potentiates 4-OHT action [29].

In this study, we demonstrate a critical prosurvival role for the IGF-1/MEK1signaling axis in breast cancer cells undergoing antiestrogen and antiprogestin treatment and uniquely demonstrate that the underlying mechanism of MEK1-mediated survival is via blockade of the proapoptotic action of the BH3-only protein BimEL.

## Materials and methods

### Cell culture

MCF-7 and T-47D ER^+ ^breast adenocarcinoma cells (early passage) were procured from the American Type Culture Collection (Rockville, MD, USA) and cultured, as previously described [17,30]. Before hormonal treatments, cells were placed in DMEM-F12 medium (Invitrogen, Carlsbad, CA, USA), supplemented with 5% dextran-coated charcoal stripped fetal bovine serum (DCC FBS; Hyclone, Logan UT, USA), 2% antibiotics-antimycotics (Invitrogen), 1% sodium pyruvate (Invitrogen) and 10 μg/ml insulin (Sigma-Aldrich, St Louis, MO, USA). For hormonal treatments, cells were seeded either in the absence or presence of insulin, allowed to adhere to the culture vessel for 16 to 24 hours, and then treated with one of the following: 10 n*M *estradiol (E2; Sigma), 10 n*M *E2 plus 1 μ*M *4-OHT in the presence or absence of 10 μ*M *MIF (Sigma Aldrich). For experiments in which cells were seeded in medium containing insulin, cells were washed with HBSS to remove insulin, before administration of hormonal treatment. As indicated in the text and figure legends, hormonal treatments also were conducted in the presence of the following agents alone or in combination: 10 μ;g/ml Insulin (Sigma),1-20 ng/ml IGF-1 (Novozymes/Gropep, North Rocks, NSW, Australia), 5 μ*M *U0126 (EMD Biosciences, Billerica, MA, USA), 25 or 50 μ;*M *PD 98059 (Calbiochem), and/or 500 μ;*M *vitamin E (Sigma Aldrich).

### Cell counts and clonogenic assay

Cells were evenly seeded in triplicate at a density to attain 50% to 70% confluence within 24 hours and treated with drugs and/or hormones, as described in the figure legends. For cell counts of the detached cell population, detached cells were collected, concentrated by centrifugation, and counted by using a hemacytometer. Adherent cells were washed twice with cold 1× PBS, trypsinized, diluted in Isoton II, and counted by using a Coulter Counter. For total cell counts, the adherent, monolayer cells were released from the culture dish by trypsinization and pooled with the detached cells collected from the medium. Before all cell counts, the cells were syringed 3 times with a 25 7/8-gauge needle to obtain single-cell suspension. Where indicated in the figure legends, trypan blue (0.08%; Sigma Aldrich) was added to the cell suspension for the identification of dead cells; trypan blue-positive cells demonstrate compromised plasma membrane integrity in dying or dead cells. Cell counts are graphed as the mean ± SD values, and statistically significant differences between treatment groups are described in the figure legends.

### Mitochondrial membrane depolarization assay

The mitochondrial depolarization assay was conducted by using the compound 5,5',6,6- tetrachloro-1,1',3,3'-tetraethyl-benzimidazolylcarbocyanine, also referred to as JC-1, according to the manufacturer's protocol (Biotium, Inc., Hayward, CA, USA) and as previously described by our laboratory [31]. All experiments were performed in triplicate. The results are expressed as the mean ± SD values, and statistically significant differences between treatment groups are described in the figure legends.

### Protein harvest, immunoblotting, and λ-phosphatase treatment

Cell lysates were harvested as described in our previous studies [13,17,30,31]. Immunoblotting was conducted according to the manufacturer's protocol by using primary antibodies to: LC3 (ab48394), p62 (ab56416), cleaved lamin A (ab52300) [Abcam]; cleaved PARP (9541), phospho-p44/42 MAP kinase (Thr202/Tyr204, 9106), total MAPK (9102), Akt (9272), phospho-Akt (Ser473, 9271), MEK1 (9124), pBim (4581), and Bim (2819) [Cell Signaling]; pERK1/2 (SC-7383), ERα (SC-8002), and IGF-1Rβ (SC-713) [Santa Cruz Biotechnology]; and β-actin (A5441) [Sigma]. Secondary antibodies included antimouse IgG (715-035-150) and anti-rabbit IgG (711-035-152) [Jackson ImmunoResearch]. Immunodetection was performed by using the ECL detection system (34080; Thermo Scientific Pierce) and HyBlot CL autoradiography film (E3012, Denville Scientific Inc., Parsippany, NJ, USA). Densitometry was used to compare signal intensity among samples by using β-actin as the loading control.

For phosphatase experiments, cell lysates were prepared and analyzed as recently described [32] in a triton-based lysis buffer with protease inhibitors, but not NaF or Na_3_VO_4_. The lysates (50 μ;g protein) were incubated for 20 minutes or 1 hour with lambda phosphatase (λ-PPase (15 μ;g/200 U) (NEB; PO753S) or calf alkaline phosphatase (CIP, 50 U) (NEB, M02909), according to the manufacturer's recommendations.

### Detection of cleaved cytokeratin 18

Evenly seeded adherent cells were treated with the drugs and/or hormones for 48, 72, and 96 hours. Detached and adherent cells were collected and lysed in ice-cold lysis buffer (10 m*M *Tris-HCl, pH 7.4/10 m*M *MgCl_2_/150 m*M *NaCl/0.5% NP-40), and the cleavage of cytokeratin 18 was measured in the cell extracts by using Peviva M30-Apoptosense ELISA, according to the manufacturer's protocol (DiaPharma, West Chester, OH, USA). Three independent experiments were performed for each treatment group. Values expressed as the mean ± SD and statistically significant differences between treatment groups are described in the figure legends.

### Reactive oxygen species (ROS) determination

MCF-7 cells (5 × 10^4 ^cells in 200 μl per well of a 96-well dish) were seeded. After 24 hours to allow cell attachment, cells were treated with the drugs and/or hormones for various times. At the end of the experimental period, the cells were washed with HBSS and loaded with 25 μ*M *5(6)-carboxy-2',7'-dichlorofluorescein diacetate (CM-H_2_DCFDA, C6827; Invitrogen) for 30 minutes. This nonfluorescent ester CM-H_2_DCFDA enters cells and is deacetylated to nonfluorescent 5-(and -6)-chloromethyl- 2',7'-dichlorodihydrofluorescein (CM-H_2_DCF) by cellular esterases. ROS rapidly oxidizes CM-H_2_DCF to the highly fluorescent 5- (and 6-) chloromethyl-2',7'-DCF (CM-DCF). After 30 minutes of incubation, intracellular ROS levels are directly proportional to CM-DCF generation. To quantify the level of intracellular CM-DCF, the cells were washed with HBSS to remove extracellular CM-DCF, treatment medium was replaced, and cells were incubated at 37°C for a short recovery period (0 to 5 minutes). CM-DCF fluorescence was measured at an excitation wavelength of 485 nm and emission at 520 nm on a fluorescent plate reader (Tecan Spectraflour Plus). Values are expressed as mean ± SD of three independent experiments, and statistically significant differences between treatment groups are described in the figure legends.

### Downregulation of MEK1 or Bim with RNA interference

RNA interference (RNAi) targeted to MEK1 or Bim (M-003571-00 and M004383-02-0020, respectively, Dharmacon) was carried out according to the manufacturer's protocol. Cells were seeded in DMEM-F12 medium containing 5% DCC FBS (antibiotic free). Twenty-four hours after seeding, cells were washed and treated with either MEK- or Bim-targeting RNAi by using Oligofectamine (Invitrogen; 12252-011). For controls, cells were treated with scrambled RNAi. Twenty-four or forty-eight hours after RNAi treatment, the cells were treated with drugs and/or hormones for various times, and harvested for either protein analysis or cell counts.

### Overexpression of MEK1 cDNAs

A MEK1-GFP plasmid expression vector [33] was purchased from Addgene (Plasmid 147461; Billerica, MA, USA), and the pEGFP-N1 parent vector, from Clonetech, 6085-1. Twenty-four hours before transfection, MCF-7 cells were seeded in DMEM-F12 medium containing 5% DCC FBS (antibiotic free) to yield approximately 50% confluence. Cells were then transfected with plasmids (4.0 μg) by using lipofectamine LTX (Invitrogen) according to the manufacturer's protocol. The transfected cell population was maintained in culture medium for 24 hours, treated with drugs for various times, and harvested for either ROS determinations, mitochondrial membrane permeabilization, or protein analysis.

### Infections with recombinant adenovirus expressing MEK1

Recombinant adenovirus [34] expressing dominant-negative MEK1 (Ad-CMV-MEK1DN, Cat 1165; Vector Biolabs) or the Ad-CMV-Null control vector (Ad-CMV, Cat 1300; Vector Biolabs) [35] was used to infect cells at an estimated multiplicity of infection of 100, which results in > 80% infection of ER^+ ^breast cancer cells. Twenty-four hours after infection, cells were treated with hormones and/or drugs for various times, and harvested for either ROS determinations, mitochondrial membrane permeabilization, or protein analysis.

### Statistical analyses

For all experiments in which data are graphed as the mean ± SD values, a minimum of three independent experiments was performed. Comparisons were made between treatment groups, and statistically significant differences were determined by one-way ANOVA by using Sigma Plot 11 for Windows, as identified in the figure legends.

## Results

### Physiologic levels of IGF-1 inhibit 4-OHT- and MIF-induced cell death by reducing the levels of oxidative stress in MCF-7 breast cancer cells

**Figure 1:**
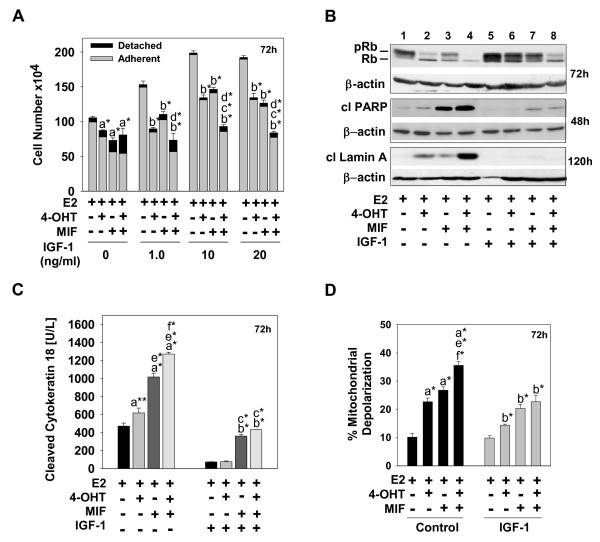
**IGF-1 attenuates the cytotoxicity of hormonal treatments in ER^+ ^breast cancer cells**. **(a) **Cell number for the adherent versus detached cell population treated with hormones in the presence or absence of various concentrations of IGF-1. **(b) **Relative levels of active, dephosphorylated Rb110 protein, designated Rb, relative to levels of the inactive, phosphorylated Rb110, designated pRb (top panel), cleaved PARP (middle panel), and cleaved lamin A (bottom panel) in cells undergoing hormonal treatments in the presence and absence of IGF-1. Protein was isolated from cells undergoing the designated treatments at 48, 72, and 120 hours, and immunoblot analysis determined the levels of Rb, pRb, cleaved PARP, and cleaved lamin A; β-actin served as a loading control. **(c) **Relative levels of cleaved cytokeratin 18 after 72 hours of the hormonal treatments in the presence and absence of IGF-1. **(d) **The percentage of mitochondrial membrane depolarization in cells undergoing hormonal treatments in the presence or absence of IGF-1. Adherent and detached cells were combined, stained with JC-1 mitochondrial membrane dye, and examined by using flow cytometry. **(a, c, d) **Data are expressed as mean ± SD (*n *= 3). Comparisons were made between treatment groups, and a statistically significant difference was identified in cell number when compared with groups treated with ^a^E2; ^b^E2 + IGF-1; ^c^E2 + 4-OHT + IGF-1; ^d^E2 + MIF + IGF-1; ^e^E2 + 4-OHT; and ^f^E2 + MIF. *Significance at *P *< 0.001.

In past studies, we reported that 4-OHT and/or MIF treatment induces MCF-7 cell detachment from the monolayer and demonstrated that the detached cells were undergoing caspase-dependent apoptosis with cleavage of PARP, lamin A, and high-molecular-weight DNA as measurable apoptotic markers [13,14,17]. These past studies were conducted in phenol red-free DMEM/F12 medium supplemented with 5% FBS that was depleted of endogenous steroid hormones through charcoal stripping. However, we now show that if hormonal treatments are conducted in this medium plus 10 μ;g/ml insulin, as recommended by the ATCC for optimal growth of MCF-7 cells, cell detachment and cell death are not detected (see Additional file 1). This concentration of insulin is supraphysiologic, and insulin at high doses can bind to and activate the IGF-1R [36]. Thus, we hypothesized that the prosurvival effects of insulin were mediated through the IGF-1R and predicted that IGF-1, at physiologic doses, would similarly attenuate the cytotoxic action of 4-OHT and/or MIF. To test this prediction, experiments were conducted in varying concentrations of IGF-1, used in combination with the hormonal treatments. These studies showed that IGF-1 at 10 and 20 ng/ml also attenuated 4-OHT- and/or MIF-induced cell death, as evidenced by a reduction in the number of detached, dying cells, while enhancing E2-mediated cell growth by approximately 50% (Figure 1a). However, cell counts did show that 4-OHT, MIF, and 4-OHT plus MIF treatments conducted in the presence of IGF-1 effectively reduced overall cell number, with the combination of 4-OHT plus MIF most effectively inhibiting cell proliferation (Figure 1a). Western blot analysis further showed predominantly the hypophosphorylated form of Rb110 in the cells treated with 4-OHT plus MIF in IGF-1-supplemented medium, in contrast to significantly higher levels of the hyperphosphorylated, inactive Rb110 present in the cells treated with either 4-OHT or MIF (Figure 1b; compare lane 8 with lanes 5 to 7). Active, dephosphorylated Rb is known to play a key role in 4-OHT- and/or MIF-induced growth arrest of ER^+ ^breast cancer cells in the G_1 _phase of the cell cycle [13,17]. So, in the presence of IGF-1, the combined treatment of 4-OHT plus MIF was able to induce cytostasis effectively, but did not appear to affect a significant death response.

Once we established that 20 ng/ml IGF-1 maximally induced cell proliferation, while blocking cell detachment (Figure 1 and data not shown), this concentration was used in all subsequent experiments, including the experiments shown in Figure 1 (b through d), in which we further characterized the IGF-1 prosurvival action. Specifically, IGF-1-treated cells showed very low levels of cleaved PARP and lamin A (Figure 1b). Further IGF-1 blocked the ability of 4-OHT and/or MIF treatment to affect the cleavage of cytokeratin 18 (Figure 1c) and depolarize the mitochondrial membrane (Figure 1d). Cytokeratin 18 cleavage, in particular, occurs during caspase-dependent apoptosis in epithelial cells and tumors derived from epithelial cells [37]. Thus, IGF-1, at physiologically relevant concentrations, blocks 4-OHT-induced and/or MIF- induced apoptotic cell death, which has been partially characterized in our previous studies and involves the activation of caspase-9, -8, and -6 [17,30].

**Figure 2:**
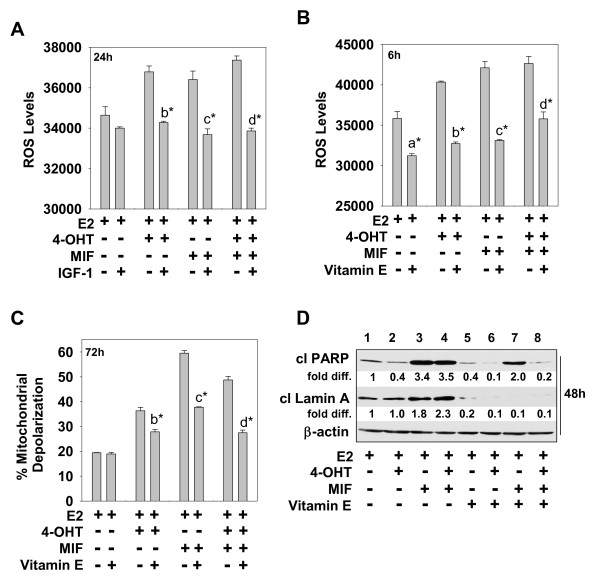
**IGF-1 attenuates 4-OHT and/or MIF-induced cell death by reducing ROS levels**. **(a, b) **ROS levels in cells undergoing hormonal treatments in the presence or absence of IGF-1 **(a) **or vitamin E **(b)**. **(c) **The percentage of mitochondrial membrane permeabilization in cells undergoing hormonal treatments in the presence or absence of vitamin E. **(d) **The levels of cleaved PARP and lamin A in cells treated with 4-OHT and/or MIF in the presence and absence of vitamin E. *fold diff*., the levels of cleaved PARP or lamin A relative to levels in E2-treated cells, which were arbitrarily assigned a value of 1.0; β-actin served as a loading control. **(a-d) **MCF-7 cells treated with E2, E2 + 4-OHT, E2 + MIF, E2 + 4-OHT + MIF in the presence or absence of IGF-1 (20 ng/ml) or vitamin E (500 μ;*M*) for the indicated times were harvested for determination of ROS levels, percentage mitochondrial membrane permeabilization, or levels of cleaved PARP and laminA, as described in Materials and Methods. Values are expressed as mean ± SD (*n *= 3). Statistically significant differences are identified between comparisons of the following treatment groups: **(a) **E2 versus E2 + vitamin E; **(b) **E2 + 4-OHT versus E2 + 4-OHT*+ *IGF-1 or E2 + 4-OHT + vitamin E; **(c) **E2 +MIF versus E2 + MIF*+*IGF-1 or E2 + MIF*+ *vitamin E; and **(d) **E2 + 4-OHT + MIF versus E2+ 4-OHT + MIF *+ *IGF-1 or E2 + 4-OHT + MIF *+ *vitamin E. *P < 0.001.

Because oxidative stress can be an upstream effector of caspase activation [38], and is suppressed by IGF-1 in breast cancer cells [39], we further determined the levels of ROS in cells undergoing the hormonal treatments in medium supplemented with or devoid of IGF-1. ROS levels were determined at various times after treatment and in multiple independent experiments. These experiments showed that ROS levels were significantly higher in cells treated with 4-OHT and/or MIF compared with E2-treated cells, but significantly reduced if IGF-1 was in the treatment medium. Figure 2a shows representative levels of ROS in cells treated with hormones for 24 hours in the presence and absence of IGF-1. The determination of ROS levels in cells harvested at earlier time points (that is, 1 hour) showed that 4-OHT plus MIF treatment induced higher levels of ROS than did either 4-OHT or MIF used as a single agent (data not shown). An essential role of ROS in mediating cell death was demonstrated by using the antioxidant vitamin E. When vitamin E was added to the treatment medium, no significant increase in ROS levels was noted in cells treated with 4-OHT and/or MIF at any time point analyzed (Figure 2b and data not shown). Further, mitochondrial membrane permeability (Figure 2c) and the cleavage of PARP and lamin A were minimally affected (Figure 2d) by hormonal therapy conducted in the presence of vitamin E. Thus, the proapoptotic actions of both 4-OHT and MIF require ROS, and the IGF-1-mediated antiapoptotic action involves a mechanism that, in large part, reduces ROS in hormonally treated breast cancer cells.

### Blockade of MEK1 activity with small-molecule inhibitors abrogates the antiapoptotic effects of IGF-1 in hormonally treated ER^+ ^MCF-7 breast cancer cells

**Figure 3:**
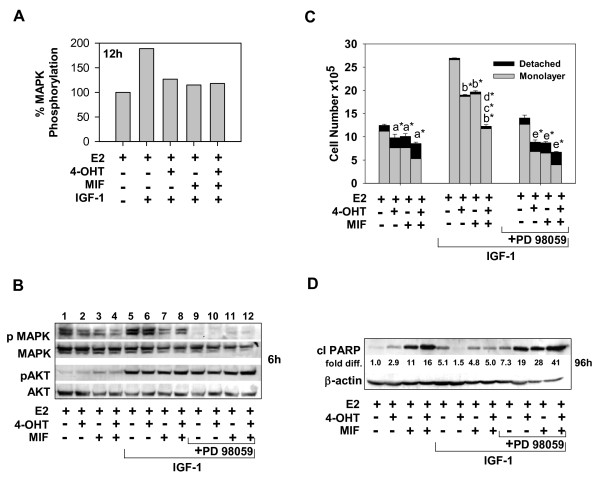
**The IGF-1/MEK signaling axis blocks the cytotoxic action of 4-OHT and/or MIF-treatments of ER^+ ^breast cancer cells**. **(a) **Graphic representation of Western blot data showing pMAPK1/2 levels in cells treated with hormones in the absence and presence of IGF-1. The pMAPK1/2 level in E2-treated cells were arbitrarily set to a value of 100%; total MAPK levels served as the loading control. **(b) **Effective and selective blockade of MAPK1/2 phosphorylation by the MEK1 inhibitor PD 98059. Total MAPK1/2 and AKT levels served as loading controls. **(c, d) **PD 98059 blocked the proliferative effects of IGF-1 and restored the ability of 4-OHT and/or MIF therapy to induce cell detachment and cleavage of PARP (apoptosis) in the MCF-7 cell populations treated with 4-OHT and/or MIF. *fold diff*., levels of cleaved PARP after correction for differences in protein loading; *β-*actin levels served as loading controls. **(a-d) **Cells were treated for the indicated time periods with hormones, as indicated in the absence or presence of IGF-1 at 20 ng/ml and/or PD 98059 at 25 μ;g/ml and harvested for immunoblotting or cell counts, as described in Materials and Methods. Values are expressed as mean ± SD (*n *= 3). Treatment effects on total cell number were determined to be significant when compared with **(a) **E2; **(b) **E2 + IGF-1; **(c) **E2 + 4-OHT + IGF-1; **(d) **E2 + MIF + IGF-1; **(e) **E2 + IGF-1 + PD 98059. *P < 0.001.

MEK1 signaling has been shown to protect against breast cancer cell death more effectively than PI3K/Akt signaling under certain cell contexts (that is, nutrient deprivation) [40]. Thus, we sought to determine whether MEK1 was a key downstream effector of the IGF-1/IGF-1R prosurvival signaling that protected MCF-7 cells from 4-OHT and/or MIF-induced death. Our read-out of MEK1 activity was the phosphorylation/activation of the mitogen-activated protein kinases MAPK1/2 that are activated by MEK1-mediated phosphorylation [41]. Under our treatment conditions and at multiple time points analyzed, cells treated with IGF-1 and E2 showed higher levels of MAPK1/2 phosphorylation (designated pMAPK) than did E2-treated or IGF-1-treated cells (Figure 3a, b; compare lane 5 with lane 1, and data not shown). Further, 4-OHT or MIF used as a single agent or in combination did lead to a significant reduction in pMAPK1/2 phosphorylation in these cells (Figure 3a). Nonetheless, at multiple time points analyzed, pMAPK1/2 in the 4-OHT- and/or MIF-treated cells was detectable by Western blot (Figure 3b, compare lanes 6 to 8 with lane 5 and data not shown), indicating that MEK1 action is only moderately suppressed by these hormonal treatments. Even when treatments were conducted in the absence of IGF-1, active, phosphorylated MAPK1/2 was detected in 4-OHT- and/or MIF-treated cells at multiple time points, including 6 hours (Figure 3b, compare lanes 2 to 4 with lane 1). Thus, MEK1 appears to be active in a large percentage of cells undergoing 4-OHT and/or MIF treatments.

To determine whether the apparent MEK1 activity imparted a growth or survival advantage to 4-OHT- and/or MIF- treated cells, we combined the small-molecule MEK1 inhibitors PD 98059 [42] or U0126 [43] with the hormonal treatments. In initial experiments, we determined that PD 98059 and U0126 effectively blocked IGF-1-and E2-induced MEK1 activity, as evidenced by the lack of detectable MAPK1/2 phosphorylation, with minimal affect on Akt phosphorylation (Figure 3b; compare lanes 9 to 12 with lanes 5 to 9 and data not shown). Importantly, PD 98059 treatment restored the basal and induced level of cell detachment (Figure 3c) and cleavage of PARP in the (Figure 3d) cell populations undergoing 4-OHT and/or MIF treatment, in addition to reducing cell proliferation in all treatment groups (Figure 3c). In a similar fashion, treatment with U0126 also blocked the proliferative (data not shown) and prosurvival effects of IGF-1 and restored the cytotoxic action of 4-OHT and MIF, which included enhancing ROS levels and increasing the percentage of mitochondrial membrane depolarization (see Additional files 2a and 2b). Treatment with vitamin E again reduced the levels of ROS, mitochondrial membrane depolarization, and cleavage of PARP and lamin A resulting from MEK1 blockade (see Additional files 2c through 2e). Considered together, these data show that small-molecule inhibitors of MEK1 effectively block the proliferative and antiapoptotic action of IGF-1 and enhance the ability of 4-OHT and MIF to induce an ROS-dependent apoptosis in ER^+ ^MCF-7 breast cancer cells.

### Blockade of MEK1 effectively induces death of MCF-7 cells with reduced IGF-1R levels

**Figure 4:**
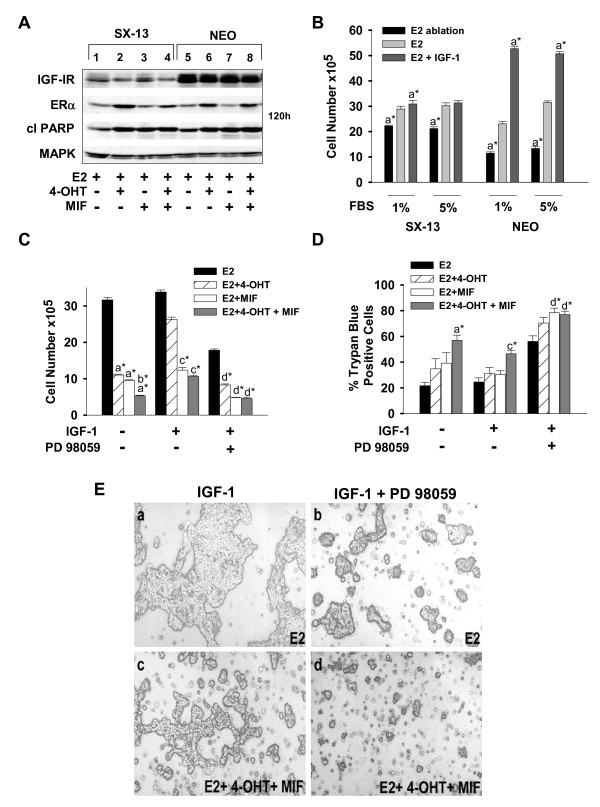
**SX13 cells expressing low-levels of IGF-1R are sensitive to the death-inducing effects of PD 98059**. **(a) **Western blot showing low levels of IGF-1R, but comparable levels of ERα and cleaved PARP in SX13 and NEO cells undergoing the indicated hormonal treatments for 120 hours. **(b, c) **Cell counts showing that IGF-1 does not enhance E2-stimulated SX13 cell proliferation, but that PD 98059 can restore the growth-inhibitory effects of 4-OHT treatment. Cells (2 × 10^5^) were seeded and, after 24 hours, treated with either 1% or 5% FBS-DCC serum in the absence (E2 ablation) or presence of E2 and/or IGF-1 for 168 hours **(b) **or 144 hours **(c)**. Cell counts were performed with a Coulter counter **(c)**. **(d) **Trypan blue exclusion assay shows that IGF-1 attenuates the death-inducing effects of 4-OHT and/or MIF treatments in an MEK1-dependent manner. At 144 hours after treatments, adherent and detached cells were collected and counted by using a hemacytometer. **(e) **Representative images show that PD 98059 effectively reduces cell number in the E2-treated cell population (compare **a **with **b**), and induces cell shrinkage and detachment, indicative of apoptosis, in the 4-OHT plus MIF-treated cell population (compare **c **with **d**). Data in (**b **through **d**) are expressed as mean ± SD (*n *= 3). The following show significant differences in the induction of apoptosis (number of detached cells) and cell proliferation for the hormonal therapies compared with: **(a) **E2; **(b) **4-OHT + MIF; **(c) **E2+IGF-1; **(d) **E2+IGF-1+PD 98059. *P < 0.05.

Both high levels of IGF-1R, as seen in MCF-7 cells, and low levels of IGF-1R are associated with a higher risk and a less-favorable clinical prognosis [44]. Thus, we wanted to determine whether IGF-1 showed similar, MEK1-dependent prosurvival effects under conditions of low-level IGF-1R expression. We analyzed a subclone of MCF-7, designated SX13, that expresses low-level IGF-1R. SX13 cells harbor the stable integration of an expression vector containing antisense to IGF-1R, whereas the parent MCF-7 cells (designated NEO) harbor the expression vector lacking the antisense [19]. IGF-1R levels in SX13 and NEO cells differ by at least twofold (Figure 4a). However, the reduction in IGF-1R does not sensitize cells to 4-OHT- and/or MIF-induced cell death. The levels of PARP cleavage in SX13 and NEO cells in response to 4-OHT and/or MIF treatment were similar (Figure 4a). Although SX13 cells were not growth-stimulated by IGF-1 above E2-stimulated growth, even under conditions of limiting serum concentration (Figure 4b) [19], IGF-1 did block the growth-inhibitory effects of 4-OHT on SX13 cells. Importantly, MEK1 blockade restored 4-OHT sensitivity in IGF-1-supplemented medium (Figure 4c). Further, IGF-1 reduced the cytotoxic action of the 4-OHT-plus-MIF combination treatment, with detectable reductions in the numbers of dead cells (trypan blue cells) (Figure 4d). When PD 98059 inhibitor was used to block MEK1 action, however, a significant increase in the numbers of trypan blue cells was seen in all the treatment groups (Figure 4d). Microscopic evaluation of SX13 and the NEO cells after 4-OHT and/or MIF treatment, in the presence and absence of IGF-1, clearly showed that PD 98059 treatment resulted in a robust reduction in cell number (data not shown, and Figure 4e, compare c and d with a and b, respectively). These studies establish that blockade of MEK1 with small-molecule inhibitors can circumvent the protective effects of IGF-1 and increase the cytotoxic, proapoptotic action of 4-OHT and/or MIF on ER^+ ^breast cancer cells with low and high levels of IGF-1R.

### MEK1 function is required to reduce ROS, which is a prerequisite of antiestrogen- and/or antiprogestin-induced cell death

**Figure 5:**
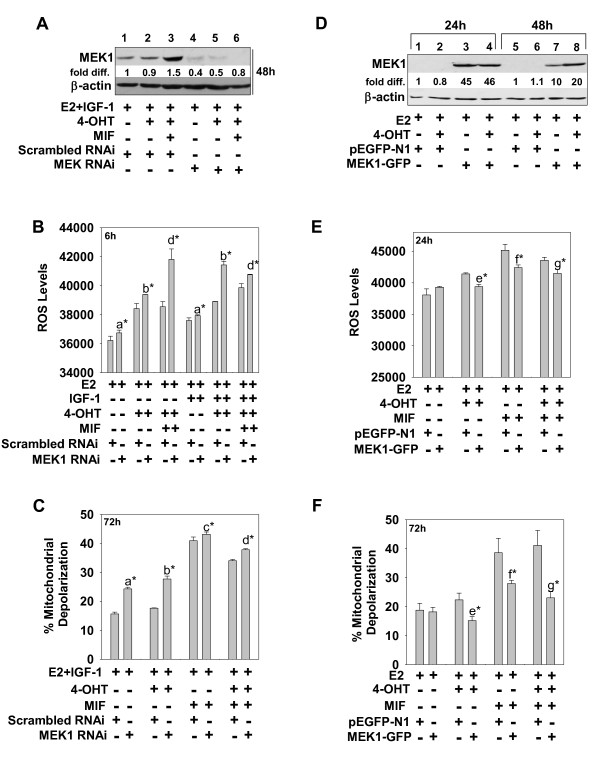
**MEK1 regulates oxidative stress and mitochondrial membrane function in ER^+ ^breast cancer cells**. **(a through c) **MEK1 downregulation blocked the prosurvival effects of IGF-1 and enhanced ROS and mitochondrial membrane depolarization in hormonally treated breast cancer cells. **(a) **Western blot shows effective RNAi targeting of MEK1, which was carried out for 48 hours before cells were treated with E2, E2 + 4-OHT, or E2 + 4-OHT + MIF for 24 hours. Protein was isolated from cells and analyzed for MEK1 expression with immunoblot analysis. **(b, c) **Cell populations with reduced MEK1 expression were analyzed at 6 and 72 hours for ROS and mitochondrial membrane depolarization, respectively. **(d through f) **MEK1 overexpression reduced ROS and mitochondrial membrane depolarization in hormonally treated breast cancer cells. Transient transfection of MEK1 cDNA (MEK1-GFP vector) increased MEK1 expression above levels seen in vector-only (pEGFP-1)-transfected control cells at 24 and 48 hours, as determined by immunoblot analysis **(d)**, and reduced ROS levels and mitochondrial membrane depolarization at 24 and 72 hours, respectively **(e, f)**. Values are expressed as mean ± SD (*n *= 3). Significant differences are designated as follows: **(a) **Scrambled versus SiMEK, E2 (± IGF-1); **(b) **Scrambled versus SiMEK, E2 + 4-OHT (± IGF-1); **(c) **Scrambled versus SiMEK, E2 + MIF (± IGF-1); **(d) **Scrambled versus SiMEK, E2 + 4-OHT + MIF; **(e) **pEGFP-N1 versus MEK1-GFP, E2 + 4-OHT; **(f) **pEGFP-N1 versus MEK1-GFP, E2 + MIF; **(g) **pEGFP-N1, E2 + 4-OHT + MIF versus MEK1-GFP, E2 + 4-OHT + MIF. *P < 0.001.

To confirm the role of MEK1 in regulating hormonally induced ROS and apoptosis, we used RNAi to downregulate MEK1 mRNA, a dominant negative, mutant MEK1 cDNA to block the action of MEK1, and a wild-type MEK1 cDNA to force MEK1 overexpression. In these experiments, targeting MEK1 expression with siRNA effectively reduced MEK1 protein levels in all treatment groups (Figure 5a, compare lanes 4 to 6 with 1 to 3). This reduction in MEK 1 expression significantly increased both the ROS levels (Figure 5b) and mitochondrial membrane depolarization (Figure 5c) in cells subjected to 4-OHT and/or MIF treatment in IGF-1-supplemented medium. Similar results were obtained when MEK1 action in MCF-7 cells was blocked by overexpression of a mutant, MEKDN (see Additional files 3a to 3c). In stark contrast, the overexpression of MEK1 wild-type cDNA, which led to detectable increases in MEK1 protein in the transfected cells (Figure 5d), reduced both the levels of ROS and mitochondrial membrane depolarization in cells undergoing 4-OHT and/or MIF treatment (Figure 5e and 5f, respectively). Thus, MEK1 overexpression in 4-OHT- and/or MIF-treated cells mimicked the prosurvival effects of IGF-1. Further, these MEK1 expression studies were consistent with the results obtained with the small-molecule inhibitors of MEK1 and confirmed a key antiapoptotic role of a MEK1-dependent pathway in MCF-7 breast cancer cells undergoing 4-OHT and/or MIF treatments.

### MEK1 blockade in antiestrogen and antiprogestin breast cancer cells induces ROS and cell death via a Bim-dependent mechanism

**Figure 6:**
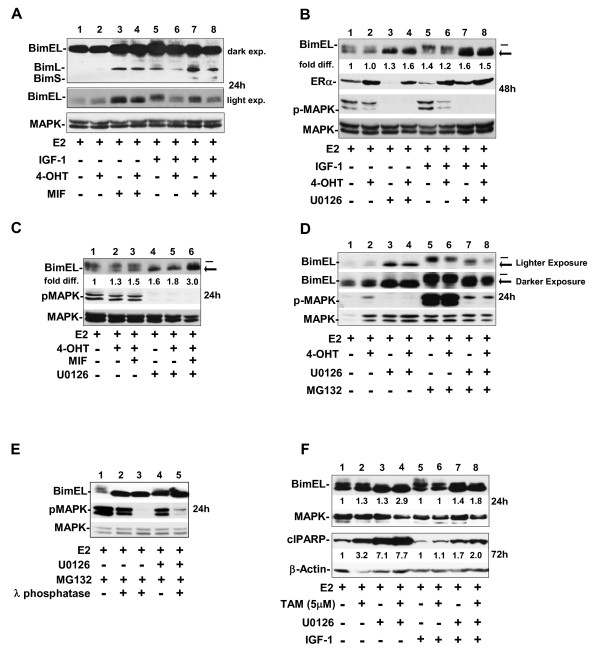
**Blockade of MEK1 increases the levels of dephosphorylated BimEL in MCF-7**. **(a through c) **Western blot shows the expression level of the Bim isoforms (EL, extra long; L, long, and/or S, short) in cells treated with hormones plus and minus IGF-1 **(a)**, with two apparent size variants of BimEL, a high-molecular-weight BimEL (top band, denoted by the dash) and a low-molecular-weight BimEL (bottom band, denoted by the arrow) **(b, c)**. *fold diff*., fold difference in signal intensity of the lower-molecular-weight BimEL band divided by the signal intensity of the high-molecular-weight BimEL band by using the total MAPK signal intensity per lane as the loading control. **(d, e) **Western blot shows the accumulation of the upper Bim EL band when cells with active MEK1 are treated with the proteasome inhibitor MG132, and preferential loss of the upper BimEL band, concomitant with accumulation of the lower-molecular-weight Bim EL form after λ protein phosphatase treatment of the protein lysates. The λ protein phosphatase digestion (described in Materials and Methods) was carried out for 20 minutes (lanes 2 and 5) or 1 hour (lane 3) on protein lysates isolated from breast cancer cells undergoing E2 treatment for 24 hours. Immunoblotting determined the levels of BimEL and pMAPK1/2; total MAPK served as loading control. **(f) **Western blot shows that TAM- and/or U0126- treated cells show significantly higher levels of BimEL protein than do E2-treated cells, with the highest levels of dephosphorylated Bim EL, correlating directly to the cleavage of PARP detectable by 72 hours.

The proapoptotic protein Bim/BOD, a member of the BH3-only group of Bcl-2 family members, is an effector of cell death on growth-factor withdrawal in many cell types, including epithelial cells [45]. Further, MEK1/MAPK1/2 signaling regulates BimEL expression via phosphorylation that facilitates BimEL degradation by the proteasome [46]. Thus, we considered Bim to be a strong candidate for the death effector mediating the cytotoxicity in hormonally treated MCF-7 cells with compromised MEK1 activity. Bim has three isoforms because of alternative splicing: BimEL, BimL, and BimS [47], with BimEL being the most abundant form expressed in MCF-7 cells under our treatment conditions (Figure 6a, top panel). BimS, which is the most cytotoxic Bim isoform and transiently expressed during apoptosis in other cell types [48], was the most difficult to detect. The BimL isoform was seen at higher levels in cells treated with MIF in comparison with E2- or 4-OHT-treated cells (Figure 6a, top panel: compare lanes 3 and 4 with 1 and lanes 7 to 8 with 5). MIF, but not 4-OHT, appeared to be inducing BimL.

Close inspection of a lighter exposure of the BimEL signal (Figure 6a, middle panel) identified a doublet band, with the upper band being the predominant form in cells treated with E2 plus IGF-1 (Figure 6a, lane 5). The lower BimEL band, which is a faster-migrating BimEL protein, was consistently detected at higher levels in cells treated with U0126 as a single agent or in combination with 4-OHT (Figure 6b; compare lanes 3 and 4 with lanes 1 and 2 and lanes 7 and 8 with lanes 5 and6), and/or MIF treatment (Figure 6c and data not shown). In addition, this lower band was often detected at higher levels than the upper BimEL band in cells treated with 4-OHT and/or MIF for 60 hours or longer in medium devoid of IGF-1 (data not shown). Overall, the relative increase in the levels of the lower BimEL band correlated to the timing of 4-OHT- and/or MIF-induced cytotoxicity in MCF-7 populations. We predicted that the lower BimEL band was the dephosphorylated form of BimEL known to be more stably maintained in cells. To determine whether phosphorylation was regulating the levels of either BimEL form, we treated cells with MG132, a commonly used proteasome inhibitor, which blocks the degradation of ubiquitinated proteins by the proteasome [49]. In these experiments, the upper BimEL protein accumulated in cells treated with MG132 plus E2, 4-OHT, and/or MIF (Figure 6i; compare lanes 5 and 6 with lanes 1 and 2 and data not shown). In contrast, in the cell populations treated with PD 98059 or U0126, the phosphorylation of BimEL was impaired and did not significantly increase after MG132 treatment (Figure 6i, compare lanes 7 and 8 with lanes 5 and 6). Further, the dephosphorylated status of the lower Bim EL band was established when protein lysates isolated from cells exposed to the different hormones were subjected to calf-intestinal-phosphatase (data not shown) or λ-phosphatase. Figure 6d shows representative results of λ-phosphatase treatments conducted for 20 minutes and 1 hour that resulted in increased levels of the lower BimEL band (Figure 6d; compare lane 1 with lanes 2 and 3, respectively). The increase in the lower BimEL band occurred with a concomitant loss of the upper BimEL band (Figure 6e; compare lane 1 with lanes 2 and 3, respectively) and was similar in size to the BimEL form generated by treatment of cells with U0126 (Figure 6e; compare lanes 2 through 4). In comparison, the λ-phosphatase treatment of protein isolated from cells treated with U0126 only modestly increased the levels of the lower BimEL band (Figure 6e; compare lane 5 with lane 4). As an internal control, the loss of pMAPK signal as a result of CIP (data not shown) and λ-phosphatase treatment was apparent in all experiments (Figure 6e; lanes 2 and 3 compared with lane 1, and lane 5 compared with lane 4). Thus, these experiments identify the lower BimEL band as the dephosphorylated from of BimEL.

Because studies of CYP2D6 polymorphisms do not clearly show that 4-OHT is a key metabolite involved in the antitumor effects of TAM treatment in patients [50], we also performed similar experiments with TAM at a dose of 5.0 μ;m, commonly used for preclinical *in vitro *studies [51]. The results of these experiments showed that IGF-1 also reduced the ability of TAM, used as a single agent or in combination with MIF, to induce cell death (Figure 6f, lane 3 compared with lane 7, and data not shown); targeting MEK1 with inhibitors led to a robust increase in the levels of dephosphorylated BimEL, with a concomitant decrease in the levels of phosphorylated BimEL; and the levels of dephosphorylated Bim EL correlated directly to the cytotoxicity of MEK1 blockade, as evidenced by increased PARP cleavage in cells by 72 hours of treatment (Figure 6f; lanes 8 and 9 compared with lanes 6 and 7).

**Figure 7:**
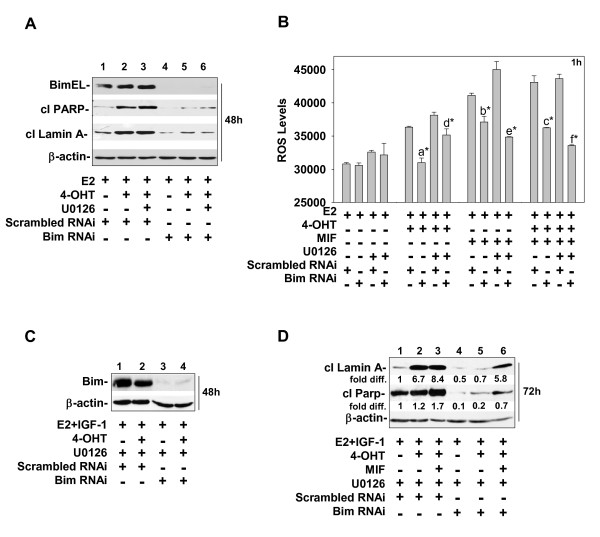
**RNAi targeting of BIM expression protects MCF-7 cells from apoptotic cell death induced by 4-OHT, MIF, and/or U0126 treatments**. **(a, c) **BIM expression was reduced in all treatment groups by RNAi targeting. MCF-7 cells were seeded in DMEM/F12 medium containing 5% DCC FBS and, after 24 hours, transfected with scrambled or BIM-targeting RNAi. Forty-eight hours after transfection, the cells were treated with E2, E2 + 4-OHT, or E2 + 4-OHT + MIF in the absence **(a) **or presence of IGF-1 (20 ng/ml) **(c, d) **and/or U0126 (5 m*M*). Cells were harvested for protein at 48 hours **(a, c**) or 72 hours **(d) **after treatment and subjected to immunoblot analysis to determine levels of Bim, cleaved PARP, and cleaved lamin **(a) **β-actin levels served as a loading control. **(b) **BIM knockdown via siRNA targeting reduces the levels of ROS generated by 1 hour of treatment with 4-OHT and/or MIF. Values are expressed as mean ± SD. Statistical significance was identified between the following treatment groups: **(a) **scrambled E2 + 4-OHT versus siBimE2 +4-OHT; **(b) **scrambled E2 + MIF versus siBim E2 + MIF; **(c) **scrambled E2 + 4-OHT + MIF versus siBim E2 + 4-OHT + MIF; **(d) **scrambled E2 + 4-OHT + U0126 versus siBim E2 + 4-OHT + U0126; **(e) **scrambled E2 + MIF + U0126 versus siBim E2 + MIF + U0126; and **(f) **scrambled E2 + 4-OHT + MIF + U0126 versus siBim E2 + 4-OHT + MIF + U0126. *P < 0.001.

To confirm that Bim played a key role in apoptotic cell death induced by antiestrogen and antiprogestin treatments when conducted in the presence and absence of MEK1 blockade, we used siRNA to downregulate Bim expression in MCF-7 cells. These experiments were performed in medium supplemented with or devoid of IGF1. The siRNA targeting of Bim was very effective in reducing BimEL protein expression when conducted in cells growing in medium devoid of IGF-1 (Figure 7a; compare lanes 4 to 6 with lanes 1 to 3). Bim downregulation under these growth conditions reproducibly attenuated the ability of 4-OHT and/or MIF, in the presence or absence of U0126, to induce the cleavage of PARP and lamin A (Figure 7a; compare lanes 4 to 6 with lanes 1 to 3). Bim downregulation also significantly reduced ROS levels in the cells treated with 4-OHT and/or MIF (Figure 7b). The ROS levels in cells treated with 4-OHT, MIF, and/or U1026 were reduced to levels present in the control E2-treated cells (Figure 7b). When IGF-1 was in the treatment medium, siRNA targeting also effectively reduced Bim levels in MCF-7 cells (Figure 7c and data not shown). The reduction in Bim expression robustly reduced the proapoptotic action of U0126 in cells of all treatment groups, but most effectively in the E2- and 4-OHT-treated cells (Figure 7d; compare lane 5 with lane 2). The siRNA data shown in Figure 7a and 7b are representative of at least three independent experiments in which cells were treated in medium devoid of IGF-1 or supplemented with IGF-1, respectively. These data provide strong evidence that Bim is a key death effector for 4-OHT- and/or MIF-induced cell death, as well as the increased cytotoxicity provided by treatment with MEK1 inhibitors

### The inherent expression level of BimEL in ER^+ ^breast cancer cells correlates with the magnitude of apoptosis induced by 4-OHT and/or MIF treatments conducted in the presence or absence of MEK1 blockade

**Figure 8:**
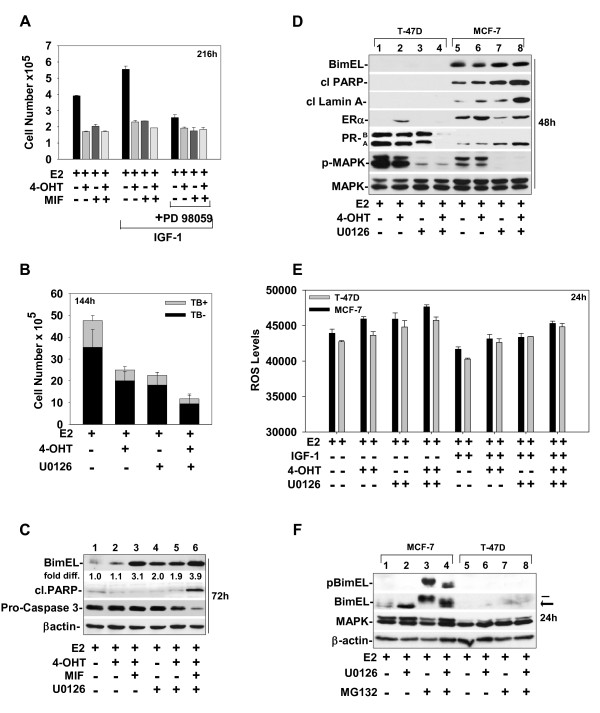
**BimEL expression levels vary between ER^+ ^breast cancer cell models and correlate to apoptotic outcome in response to hormonal treatments and MEK1 blockade**. **(a, b) **Cell-number determinations showed that IGF-1 stimulated T-47D cell growth via a MEK1-dependent proliferation pathway. T-47D cells were treated with the indicated hormones in the presence or absence of IGF-1 (20 ng/ml) plus and minus PD 98059 for 216 hours **(a) **or U0126 for 144 hours **(b)**. **(c) **Western blot showed BimEL levels relative to the levels of cleaved PARP in T-47D cells treated with hormones in the absence or presence of U0126 for 72 hours. The levels of ER and PR are provided for validation of the ER and PR status of T-47D and MCF-7 cells used in this study. **(d) **Western blot compared the levels of BimEL in MCF-7 versus T-47D cells treated with hormones plus or minus U0126 for 48 h. **(e) **ROS levels were determined for T-47D and MCF-7 cells treated with the indicated hormones in the presence or absence of IGF-1 plus or minus MEK1 blockade with U0126. **(f) **Western blot showed that treatment with MG132 caused an accumulation of phosphorylated Bim EL in MCF-7 cells, but not in T-47D cells. **(a **through **f) **As described in Materials and Methods, at the indicated times, cells were harvested and analyzed either for cell counts **(a, b)**, protein expression by SDS/PAGE and immunoblotting for BimEL, pBimEL, pro-caspase-3, pMAPK, and total MAPK or β-actin, which were used as loading controls **(c, d)**, or for ROS determination **(e)**.

T-47D is an independent breast cancer cell model that expresses ER and PR and is commonly used for studies analyzing the effects of antiestrogen blockade of ER function [17,30,31]. In comparison to MCF-7 cells, T-47D cells show lower basal levels of BimEL, and this reduced level of BimEL expression has been correlated to a reduced level of paclitaxel-induced apoptosis [52]. Thus, we characterized the level and phosphorylation status of BimEL relative to the induction of cytostasis and cytotoxicity in T-47D cells by 4-OHT and/or MIF treatment in the presence and absence of IGF-1 and under conditions of MEK1 blockade. Cell counts showed that IGF-1 stimulated T-47D cell growth above proliferation levels seen in the E2-treated population and that PD 98059 effectively reduced the IGF-1-mediated proliferation (Figure 8a). However, no detectable increase was seen in the number of trypan blue cells within T-47D cell populations as a result of any of the treatments, even after extended periods of treatment (that is, 144 hours) (Figure 8b and data not shown). By 72 hours of treatment, cleavage of PARP could be detected in T-47D cells treated with 4-OHT plus MIF plus U0126 (Figure 8c; compare lane 6 with lanes 1 to 5), concomitant with a reduction in the levels of procaspase 3, indicative of its activation. Cleavage of PARP and lamin A was detected in cells treated with MIF, 4-OHT plus MIF, and 4-OHT plus U0126, but only at later time points and at modest levels (data not shown). The 4-OHT-treated T-47D cells never showed evidence of apoptotic cell death; neither cleavage of PARP nor that of lamin A was detected in 4-OHT-treated cells, even after 216 hours of treatment. In comparison with the cytotoxic effect on MCF-7 cells, T-47D cells appeared essentially resistant to apoptosis, with absence of cleavage of PARP and lamin A in T-47D cells treated with 4-OHT and/or MIF in the absence or presence of U0126 for 48 hours (Figure 8d; compare lanes 1 to 4 with lanes 5 to 8, respectively). The reduced ability of T-47D cells to undergo apoptotic cell death correlated to an approximate twofold lower level of basal BimEL expression in T-47D cells compared with MCF-7 cells. This difference can readily be seen in Figure 8d, in which equal loading of lysates shows no detectable level of Bim EL in T-47D cells compared with readily detectable Bim EL expression in MCF-7 cells (Figure 8d; compare lanes 5 to 8 with lanes 1 to 4). ROS levels in 4-OHT- and/or MIF-treated T-47D also were less than those induced in MCF-7 cells (Figure 8e and data not shown).

**Figure 9:**
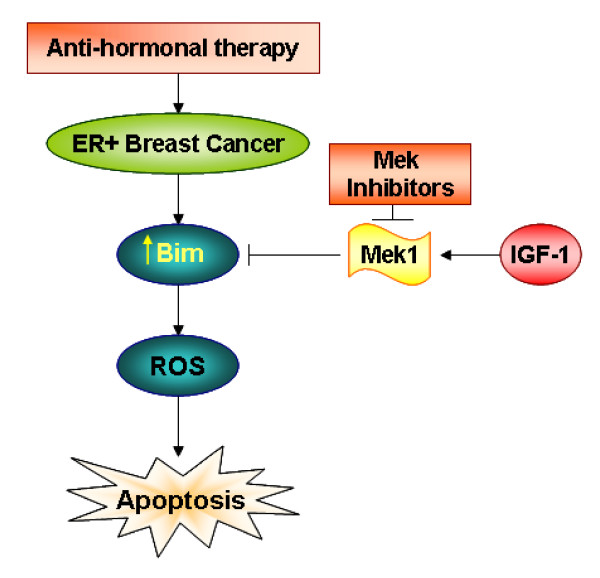
**A schematic representation of the role of BimEL and induction of apoptosis in ER^+ ^breast cancer cells**. This model is a summary of data showing that MEK1 blockade, in addition to hormonal treatment (antiestrogen or antiprogestin treatment) will activate Bim via dephosphorylation and induce an ROS-dependent apoptotic cell death in some ER^+ ^breast cancer cells, particularly if IGF-1-induced IGF-IR signaling is active.

Although apoptotic death was minimally induced in T-47D cells, treatment with U0126 effectively reduced the levels of pMAPK1/2 in T-47D cells, which were inherently higher than pMAPK1/2 levels in MCF-7 cells (Figure 8d). Because pMAPK1/2 levels were at least twofold higher in T-47D cells than in MCF-7 cells, we performed experiments with MG132 to determine whether the intrinsic turnover rate of BimEL was higher in T-47D cells than in MCF-7 cells. MG132 treatment did not increase the intracellular levels of BimEL in T-47D cells (Figure 8f; compare lanes 7 and 8 with lanes 3 and 4). These data show that the basal level of BimEL expression can vary between ER^+ ^breast cancer cell models by mechanisms independent of MEK1/MAPK12-mediated phosphorylation and proteasomal turnover. Thus, MEK1 targeting may be effective only in ER^+ ^breast cancer cells with high intrinsic levels of BimEL (schematically summarized in Figure 9).

## Discussion

In this study, we provide evidence that an improved treatment approach for ER^+ ^breast cancer could be the use of antiestrogen and/or antiprogestin therapy in combination with the targeted blockade of the dual-specificity MEK1 kinase. Specifically, this study used a variety of expression vectors, siRNA targeting, and small-molecule inhibitors of MEK kinase to demonstrate the following key data: (a) physiologic levels of IGF-1 protect ER^+ ^breast cancer cells from antiestrogen- and antiprogestin-induced cell death through an MEK1-dependent mechanism; (b) MEK1 activation blocks ROS induction and/or accumulation that is required for antiestrogen- and antiprogestin-induced apoptotic cell death; and (c) MEK1 blockade circumvents IGF-1-mediated protection and induces a Bim-dependent, ROS-mediated apoptotic cell death in antiestrogen- and/or antiprogestin-treated breast cancer cells.

Our studies are based on the hypothesis that targeting PR along with ER should more effectively reduce breast cancer cell growth than does treatment with an antiestrogen, because progesterone, like estrogen, is mitogenic in the breast [53] and drives mammary tumor proliferation in multiple model systems. Consistent with a mitogenic role for PR in breast cancer, an *in vivo *preclinical study [54,55] recently showed that MIF treatment actually prevented the development of mammary carcinogenesis in mice carrying a mutated *BRCA1 *gene [55]. Thus, targeting the PR with an antiprogestin like MIF along with antiestrogen therapy should have added benefit for all ER^+ ^breast cancer patients, and particular benefit for patients with ER^+^, antiestrogen-unresponsive tumors. For example, blockade of the PR may be quite effective for the subpopulation of ER^+ ^breast cancers identified by Fuqua and colleagues [8] that are PR-A-rich and show a very poor disease-free survival rate after antiestrogen therapy. The fact that MIF treatment is well tolerated and can block breast epithelial cell proliferation in premenopausal women [12] lends further support for MIF or other antiprogestins currently being developed [11,54] to be used in combination with antiestrogen therapy. To date, only three clinical trials have been conducted with MIF. In these trials, MIF was used as a monotherapy [reviewed in [9]], and two of the trials showed efficacy of MIF monotherapy similar to that of TAM therapy against metastatic breast cancer.

In support of targeting both ER and PR as a treatment approach to breast cancer, our past studies demonstrated that 4-OHT and MIF more effectively induce growth arrest and cell death than do either 4-OHT or MIF treatment of ER^+^PR^+^, antiestrogen-sensitive [13,14], and ER^+^PR^+^, antiestrogen-resistant breast cancer cells [17]. Improved efficacy was also seen when the antiestrogen ICI 182, 780 (faslodex) was combined with MIF [17]. Previous *in vivo *studies with human breast cancer xenografts in nude mice determined that TAM-plus-MIF combined treatment effected a more-robust antitumor response than did TAM or MIF [11]. This study still continues to support the concept of using TAM-plus-MIF combination therapy because this combined treatment induced a robust cytostatic response in ER^+ ^breast cancer cells treated in medium supplemented with IGF-1, even though the cytotoxic effects of the combined treatment were markedly attenuated by IGF-1. Overall, IGF-1 appears to convert hormonally induced cytotoxicity to a cytostatic outcome. Because cytostasis is not a terminal state, breast cancer cells treated in the presence of IGF-1 could potentially escape antiestrogen- and/or antiprogestin-induced cytostasis via genetic or epigenetic changes that lead to the development of resistance. Thus, the use of an antiestrogen with an antiprogestin may not completely alleviate problems of resistance, particularly in patients with high circulating levels of IGF-1 [56].

Combining a MEK1 inhibitor with antiestrogen and/or antiprogestin treatment very effectively blocked the proliferative and antiapoptotic effects of IGF-1 in MCF-7 cells. Thus, MEK1 appears to be a key to breast cancer cell survival and proliferation. A critical prosurvival role of MEK1 in breast cancer cells is supported by elegant studies from the Eastman laboratory, which demonstrated a more critical role of MEK1/MAPK signaling in breast cancer cell survival than that of Akt signaling [40]. Our study, however, is quite distinct from the study by the Eastman laboratory, which did not use hormonal therapy, or identify the key role of the proapoptotic BimEL protein in mediating death in response to MEK1 blockade in hormonally treated breast cancer cells. In more recent studies, a prosurvival role for MEK1 in blocking the cytotoxicity of TNF-α against MCF-7 cells has also been demonstrated [57]. Thus, recognition of an important role is growing for MEK1-mediated signaling in breast cancer cell survival.

Not all published studies concur with a key prosurvival role for MEK1 in hormonally treated breast cancer cells. For example, Dufourny *et al. *[58] reported that mitogenic signaling induced by IGF-1 in MCF-7 human breast cancer cells was independent of the mitogen-activated protein kinases (MAPK1/2) and that PD 98059 was unable to restore antiestrogen efficacy. In their study, PI3-K-induced signaling mediated survival. We believe that one explanation for inconsistencies in the reported role(s) of MEK1 versus that of AKT is the potential variation in MCF-7 cell lines between laboratories. This variation can result for a number of reasons, including the length of passage of the MCF-7 cells; (that is, early versus late passage [59]) and the fact that an inherent clonal heterogeneity within the MCF-7 cell line itself [17] can easily result in the selection of cells with the fastest proliferation rates. The MCF-7 cells used in this study were cultured from early-passage MCF-7 cells (ATCC), still maintain inducible MEK/MAPK signaling, and do not show constitutive PI3K/Akt signaling. However, a recent study in lung cancer cells demonstrated that constitutive AKT expression reduced the level of BimEL expression to such an extent that, even with MEK1 blockade, apoptosis was not induced [60]. So it will be important to investigate how constitutive Akt activation affects the IGF-1/MEK1 prosurvival axis described in this study.

Of particular importance, this study provides strong evidence that not merely the levels of BimEL in cells determine a cytotoxic outcome. More important, it appears that the conversion of phosphorylated BimEL to the dephosphorylated form is a key to the BimEL proapoptotic action. Nonetheless, the intrinsic level of BimEL expression is important, as seen by the studies using the T-47D breast cancer cells. We show that T-47D cells express lower levels of basal BimEL protein and do not readily undergo hormonally induced apoptotic cell death, even when cells are treated with an MEK1 inhibitor. So, targeting MEK1 may not yield optimal BimEL-induced apoptosis in all breast cancer patients undergoing endocrine therapy for ER^+^, luminal-type breast cancers. To identify the breast cancer patients that will benefit from MEK1 targeting, it will be important to determine the various mechanisms regulating BimEL expression and function in T-47D cells and in other breast cancer cell models that express low levels of Bim. To this end, our current studies are aimed at understanding the multiple pathways that modulate BimEL expression and function in different ER^+ ^breast cancer cell models. With the knowledge that BimEL can affect death in ER^+ ^breast cancer cells treated with antiestrogens, it is interesting to speculate that the overexpression of Bcl2 that has been identified in antiestrogen-resistant sublines and breast cancers [61] may be selected, in part, by the cancer cell survival being dependent on blocking the cytotoxic action of BimEL, as Bcl2 binding to BimEL can abrogate the BimEL ability to induce apoptosis [62].

Although not a main focus of this article, the prosurvival effects of vitamin E (α-tocopherol) described in this study should be noted. Vitamin E effectively blocked apoptosis induced by 4-OHT and MIF, in the absence and presence of MEK1 blockade. Vitamin E treatment specifically reduced ROS in cells undergoing these treatments. A recent study from the Poirot laboratory [63] similarly showed that vitamin E blocked TAM induced breast cancer cell death by inhibiting the production of oxysterols and ROS. It does appear that at least part of the cytotoxic action of TAM, 4-OHT, and other SERMs results from their binding to high-affinity microsomal antiestrogen binding sites (AEBS), which alters cholesterol metabolism in such a manner that oxysterols and ROS accumulate in breast cancer cells [63-65]. Whether the increased ROS generated due to MEK1 blockade somehow results from a similar impairment of cholesterol metabolism remains to be determined. However, it is clear that vitamin E blocks the ROS induction/accumulation that results from MEK1 blockade during antiestrogen and/or antiprogestin treatments and that the abrogation of ROS blocks breast cancer cell death. From these data, it is tempting to speculate that breast cancer patients undergoing antiestrogen therapy may benefit from a diet low in vitamin E. Minimally, further studies are needed the better to define the mechanism of action of vitamin E, its effect on the MEK1/MAPK prosurvival axis that contributes to the regulation of the Bim proapototic action, and its effect on the efficacy of endocrine therapy for breast cancer.

In breast cancer tissue from patients, the downregulation of Bim expression has been associated with breast cancer progression, in conjunction with downregulation of SIAH1 expression [66]. However, we are unaware of any studies analyzing Bim expression levels relative to endocrine efficacy in patients. Interestingly, Butt and colleagues [51] recently reported that PUMA levels in a small cohort of breast cancer patients predict patient outcome and tamoxifen responsiveness. PUMA, like Bim, is a BH3-only protein of the Bcl2 family of proteins and an apoptotic regulator. PUMA downregulation was shown to mediate an apoptotic response to TAM in human breast cancer cells, but manipulation of PUMA levels alone was unable to ameliorate completely TAM-induced apoptosis [51]. Butt and colleagues proposed that there is a "complex interplay between numerous apoptotic regulators in coordinating the cytotoxic, endocrine response." Our data are in full agreement with this prediction and support the conclusion that dephosphorylated Bim EL will be one of the apoptotic regulators important in predicting endocrine response. An already-known interplay exists between Bim and Puma proteins in regulating taxane-induced cell death in breast cancer cells. In this response, PUMA displaces Bim from binding Bcl2, so Bim is free to affect negatively the mitochondrial integrity and execute its proapoptotic function [67]. Our study, combined with these recent studies, allow us to predict that the regulation of Bim, along with PUMA in breast cancer cells, will be pivotal to their response to hormonal therapy and some chemotherapies.

## Conclusions

This study has identified the IGF-1/IGF-IR/MEK prosurvival axis that exists in ER^+ ^breast cancer cells to attenuate significantly the cytotoxic action of antiestrogen and antiprogestin treatment, with little effect on the antiproliferative action of these hormones. We further identified BimEL as the death effector being regulated by the IGF-1/IGF-IR/MEK prosurvival axis. Targeting MEK1 in conjunction with hormonal therapy as an initial treatment option would be a new approach and should be considered, because the recurrence of breast cancer in women receiving SERMs is still a major clinical challenge, and a large number of ER^+ ^breast cancers are initially resistant to antiestrogen therapy, possibly due to IGF-1-mediated survival effects [56].

## Abbreviations

4-OHT: 4-hydroxytamoxifen; ER: estrogen receptor; IGF-1: insulin-like growth factor-1; IGF-1R: insulin-like growth factor receptor; MAPK: mitogen-activated protein kinase; MEK1DN: MEK1-dominant negative kinase; MIF: mifepristone; cl PARP: cleaved poly-ADP ribose polymerase; PR: progesterone receptor; Rb: retinoblastoma protein; ROS: reactive oxygen species; TAM: tamoxifen.

## Competing interests

The authors declare that they have no competing interests.

## Authors' contributions

PVS conceived the study. PVS, SPT, DL, MM, SH, and MT participated in the experimental design, data interpretation, and manuscript preparation. SPT, ST, MRD, WHJ, AEW, AS, and PVS performed and analyzed cell-proliferation/death assays, immunoblotting, and RNAi targeting experiments. SPT, ST, MT, and PVS analyzed data and performed statistical analysis. All authors read and approved the final manuscript.

## Supplementary Material

Additional file 1 **Insulin protects ER^+ ^breast cancer cells from 4-OHT and MIF-induced cytotoxicity**. MCF-7 cells were treated with hormones in the presence or absence of insulin (10 mg/ml). At various times of treatment, representative live images of cells were captured by using phase-contrast microscopy **(a)**; adherent versus detached cells were counted **(b)**; or cells were harvested for protein, which was analyzed with immunoblotting to determine levels of cleaved PARP **(c)**, which is a marker of apoptosis in MCF-7 cell populations. Click here for file

Additional file 2 **U0126, a selective inhibitor of MEK1, induces ROS-dependent apoptosis in MCF-7 cells undergoing hormonal treatments in the presence or absence of IGF-1**. **(a, b) **Cells treated with hormones in the presence of IGF-1 (20 ng/ml) plus U0126 (5 m*M*; 30 minutes pretreatment) versus treatments conducted in the absence of U0126 showed significant increases in ROS levels and mitochondrial depolarization. **(c **through **e) **However, the increased ROS levels, mitochondrial membrane permeabilization, and increased levels of cleaved PARP and lamin A (markers of apoptosis) were significantly reduced if cells were pretreated with vitamin E (500 μ;*M*, 30-min pretreatment) under all treatment conditions. Click here for file

Additional file 3 **Overexpression of a MEK1 dominant negative mutant protein (MEKDN) induces ROS-dependent apoptosis in MCF-7 cells undergoing hormonal treatments in the presence of IGF-1**. In multiple independent experiments, MCF-7 cells infected with Ad-CMV-MEK1DN for various time periods showed statistically significant increases in ROS levels **(a, b)**, and in the percentage of mitochondrial membrane depolarization in the infected MCF-7 cell populations, as compared with MCF-7 cells infected with Ad-CMV control vector **(c) **. Click here for file
