# Supplementary material for: Insulin-like growth factor 1 attenuates antiestrogen- and antiprogestin-induced apoptosis in ER+ breast cancer cells by MEK1 regulation of the BH3-only pro-apoptotic protein Bim
Source: Breast Cancer Res. 2012 Mar 19;14(2):R52. doi: 10.1186/bcr3153 (PMC3446386; doi:10.1186/bcr3153)
Supplement: Additional file 1 — Insulin protects ER+ breast cancer cells from 4-OHT and MIF-induced cytotoxicity. MCF-7 cells were treated with hormones in the presence or absence of insulin (10 mg/ml). At various times of treatment, representative live images of cells were captured by using phase-contrast microscopy (a); adherent versus detached cells were counted (b); or cells were harvested for protein, which was analyzed with immunoblotting to determine levels of cleaved PARP (c), which is a marker of apoptosis in MCF-7 cell populations. [file bcr3153-S1.PDF]

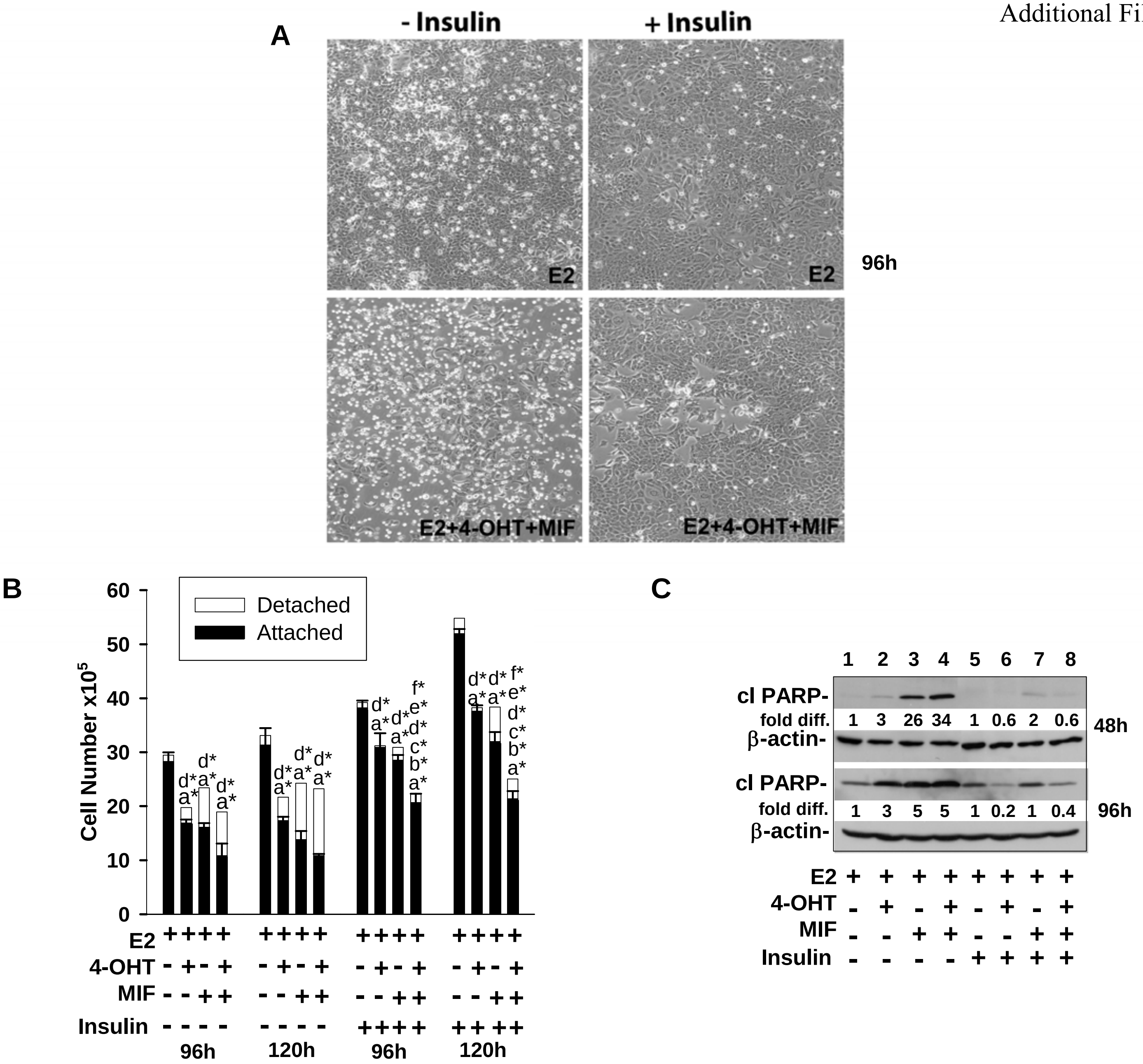

Additional File 1. Insulin protects ER+ breast cancer cells from 4-OHT and MIF-induced cytotoxicity. (A-B) Insulin blocks 4-OHT- and/or MIF-induced cell detachment. MCF-7 cells were treated in the presence or absence of insulin (10 µg/ml) with E2, E2 + 4-OHT, E2 + MIF , and E2 + 4-OHT + MIF. At 96 h treatment, representative live images of cells were captured at 200 x magnification using phase contrast microscopy and showed a high level of cell detachment in cells treated with 4-OHT plus MIF therapy **(A)**. At the times indicated, adherent monolayer cells were harvested and counted using a Coulter counter. Detached cells in the culture medium were collected, concentrated by centrifugation, and counted using a hemacytometer. Cell number is the sum of the detached and monolayer cells **(B)**. **(C)** Insulin blocks 4-OHT- and/or MIF- induced cell death. At the indicated times, cells were collected and protein lysates prepared for SDS/PAGE and immunoblotting to determine levels of cleaved PARP relative to β-actin levels which served as the loading control. Data shown is representative of at least three independent experiments. Data in **(B)** are expressed as mean ± S.D. (n=3). The following designations show significant differences in adherent cell number for treatments compared to: [a] E2 +/- Insulin, [b]4-OHT +/- Insulin; or [c]MIF +/- Insulin; and significant difference in detached cell number for treatments compared to: [d]E2 +/- Insulin, [e]4-OHT +/- Insulin, and [f]MIF +/- Insulin. The symbol * denotes significance at $P < 0.05$.
