# Supplementary material for: Insulin-like growth factor 1 attenuates antiestrogen- and antiprogestin-induced apoptosis in ER+ breast cancer cells by MEK1 regulation of the BH3-only pro-apoptotic protein Bim
Source: Breast Cancer Res. 2012 Mar 19;14(2):R52. doi: 10.1186/bcr3153 (PMC3446386; doi:10.1186/bcr3153)
Supplement: Additional file 2 — U0126, a selective inhibitor of MEK1, induces ROS-dependent apoptosis in MCF-7 cells undergoing hormonal treatments in the presence or absence of IGF-1. (a, b) Cells treated with hormones in the presence of IGF-1 (20 ng/ml) plus U0126 (5 mM; 30 minutes pretreatment) versus treatments conducted in the absence of U0126 showed significant increases in ROS levels and mitochondrial depolarization. (c through e) However, the increased ROS levels, mitochondrial membrane permeabilization, and increased levels of cleaved PARP and lamin A (markers of apoptosis) were significantly reduced if cells were pretreated with vitamin E (500 μ;M, 30-min pretreatment) under all treatment conditions. [file bcr3153-S2.PDF]

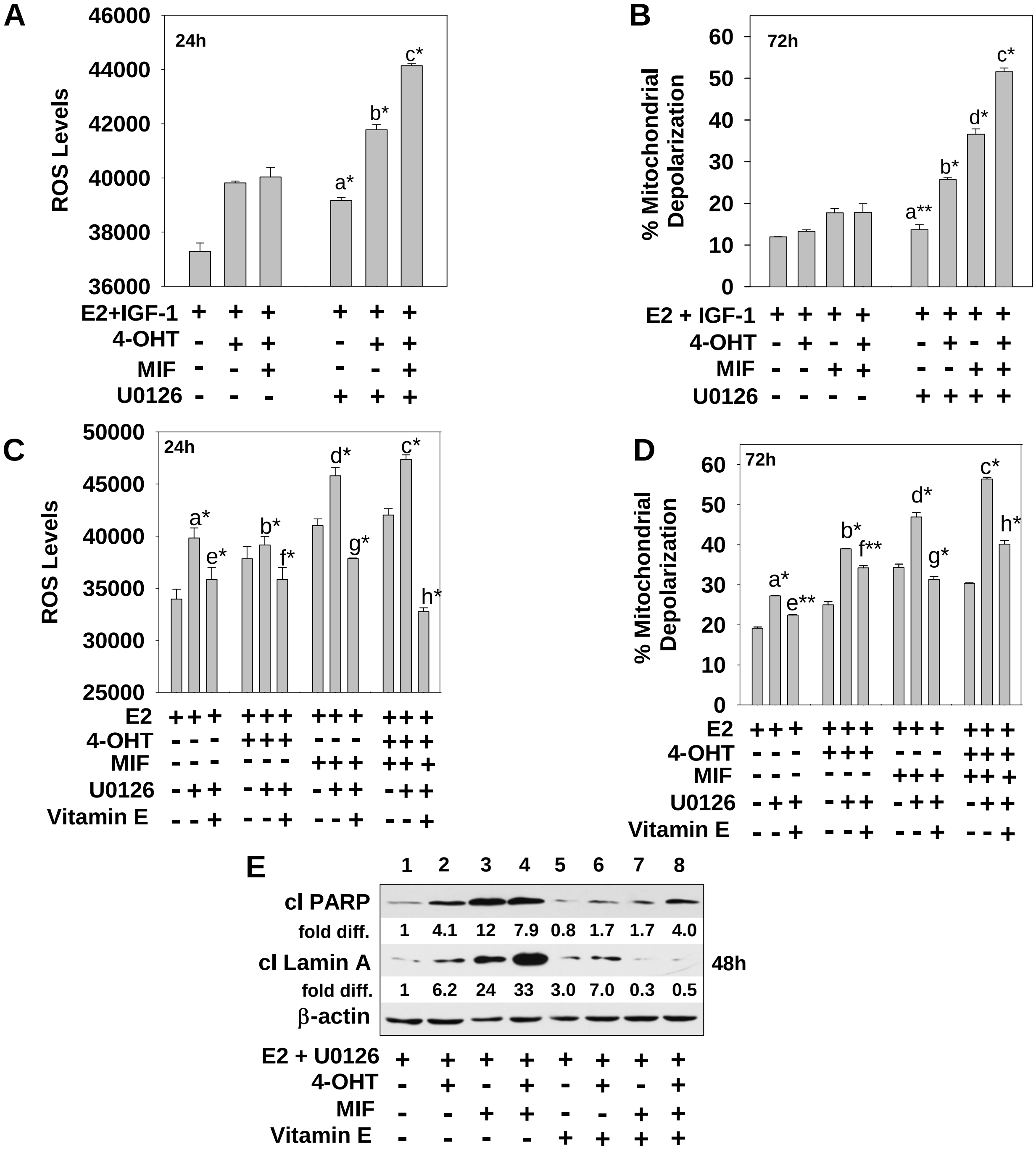

Additional File 2. U0126, a selective inhibitor of MEK1, induces ROS-dependent apoptosis in MCF-7 cells undergoing hormonal treatments in the presence or absence of IGF-1. (A-B) U0126 (5μM; 30 min pretreatment) significantly enhanced ROS levels **(A)** and the percent of mitochondrial depolarization **(B)** in cells treated with hormones in the presence of IGF-1 (20ng/ml). **(C-E)** Vitamin E (500 uM, 30 min pretreatment) blocked 4-OHT- and/or MIF- induced ROS **(C)**, and reduced the percent of mitochondrial membrane depolarization **(D)** and cleavage of PARP and lamin A **(E)** in cells treated in medium supplemented with E2 +/- U0126 (5μM; with 30 min pretreatment). *fold diff.*, the increase in signal intensity of cleaved PARP or lamin A relative to the signal intensity in E2 + U0126-treated cells (lane 1), arbitrarily set to a value of 1.0; corrections for loading were based on β-actin signal intensity per sample. Values are expressed as mean ± S.D. Significant differences between treatments are designated as follows: [a] E2 versus E2 + U0126 (+/-IGF-1); [b] E2 + 4-OHT versus E2 + 4-OHT + U0126 (+/-IGF-1); [c] E2 + 4-OHT+ MIF versus E2 + 4-OHT + MIF + U0126 (+/-IGF-1); [d] E2 + MIF versus E2 + MIF + U0126 (+/-IGF-1); [e] E2 + U0126 (+/- vitamin E); [f] E2 + 4-OHT + U0126 (+/- vitamin E); [g]E2 + MIF + U0126 (+/-vitamin E); [h]E2 + 4-OHT + MIF + U0126 (+/- vitamin E) The symbol * and ** represent statistical significance at $P<0.001$ and $P<0.05$, respectively.
