# Supplementary material for: Insulin-like growth factor 1 attenuates antiestrogen- and antiprogestin-induced apoptosis in ER+ breast cancer cells by MEK1 regulation of the BH3-only pro-apoptotic protein Bim
Source: Breast Cancer Res. 2012 Mar 19;14(2):R52. doi: 10.1186/bcr3153 (PMC3446386; doi:10.1186/bcr3153)
Supplement: Additional file 3 — Overexpression of a MEK1 dominant negative mutant protein (MEKDN) induces ROS-dependent apoptosis in MCF-7 cells undergoing hormonal treatments in the presence of IGF-1. In multiple independent experiments, MCF-7 cells infected with Ad-CMV-MEK1DN for various time periods showed statistically significant increases in ROS levels (a, b), and in the percentage of mitochondrial membrane depolarization in the infected MCF-7 cell populations, as compared with MCF-7 cells infected with Ad-CMV control vector (c) . [file bcr3153-S3.PDF]

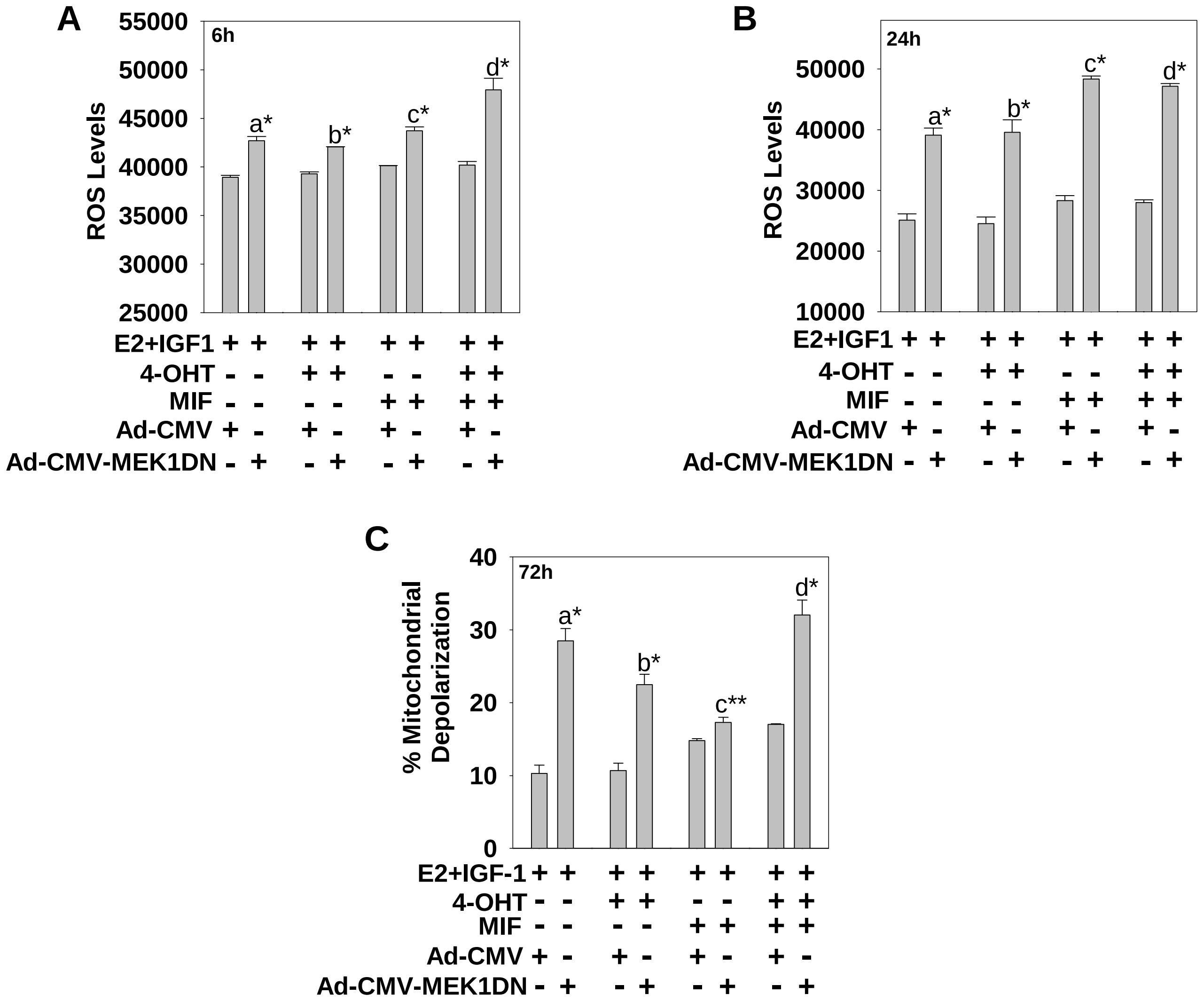

Additional File 3: Overexpression of a MEK1 dominant negative mutant protein (MEKDN) induces ROS-dependent apoptosis in MCF-7 cells undergoing hormonal treatments in the presence or absence of IGF-1. (A-B) ROS levels were determined for MCF-7 cells infected with Ad-CMV alone or Ad-CMV-MEK1DN. **(C)** The percent of mitochondrial membrane depolarization was determined for MCF-7 cells infected with Ad-CMV or Ad-CMV-MEKDN. **(A-C)** Cells were infected with either Ad-CMV alone or Ad-CMV-MEK1DN for 24 h, after which cells were treated with E2, E2 + 4-OHT, E2 + MIF, or E2 + 4-OHT + MIF in presence of IGF-1 (20ng/mL). The cells were harvested for ROS determination or mitochondrial membrane depolarization levels as described in materials and methods at 6 h **(A)** and 24 h **(B)** and 72 h **(C)**. The Values are expressed as mean ± S.D (n=3). Statistical significance was determined by One-Way ANOVA using sigma plot 11 for windows. Significant differences are indicated between treatment groups as follows: [a] E2 + IGF-1 (Ad-CMV- versus Ad-CMV-MEK1DN-infected); [b] E2 + 4-OHT + IGF-1 (Ad-CMV- versus Ad-CMV-MEK1DN-infected; [c] E2 + MIF + IGF-1 (Ad-CMV- versus Ad-CMV-MEK1DN-infected); [d] E2 + 4-OHT + MIF + IGF-1 (Ad-CMV- versus Ad-CMV-MEK1DN-infected). The symbol * represent statistical significance at P<0.001.
